# Differentially expressed galactinol synthase(s) in chickpea are implicated in seed vigor and longevity by limiting the age induced ROS accumulation

**DOI:** 10.1038/srep35088

**Published:** 2016-10-11

**Authors:** Prafull Salvi, Saurabh Chandra Saxena, Bhanu Prakash Petla, Nitin Uttam Kamble, Harmeet Kaur, Pooja Verma, Venkateswara Rao, Shraboni Ghosh, Manoj Majee

**Affiliations:** 1 Lab 203, National Institute of Plant Genome Research, Aruna Asaf Ali Marg, New Delhi 110067, India

## Abstract

Galactinol synthase (GolS) catalyzes the first and rate limiting step of Raffinose Family Oligosaccharide (RFO) biosynthetic pathway, which is a highly specialized metabolic event in plants. Increased accumulation of galactinol and RFOs in seeds have been reported in few plant species, however their precise role in seed vigor and longevity remain elusive. In present study, we have shown that galactinol synthase activity as well as galactinol and raffinose content progressively increase as seed development proceeds and become highly abundant in pod and mature dry seeds, which gradually decline as seed germination progresses in chickpea (*Cicer arietinum*). Furthermore, artificial aging also stimulates galactinol synthase activity and consequent galactinol and raffinose accumulation in seed. Molecular analysis revealed that GolS in chickpea are encoded by two divergent genes (*CaGolS1* and *CaGolS2*) which potentially encode five CaGolS isoforms through alternative splicing. Biochemical analysis showed that only two isoforms (CaGolS1 and CaGolS2) are biochemically active with similar yet distinct biochemical properties. CaGolS1 and *CaGolS2* are differentially regulated in different organs, during seed development and germination however exhibit similar subcellular localization. Furthermore, seed-specific overexpression of *CaGolS1* and *CaGolS2* in *Arabidopsis* results improved seed vigor and longevity through limiting the age induced excess ROS and consequent lipid peroxidation.

Synthesis of Raffinose Family of Oligosaccharides (RFOs) is a highly specialized metabolic event in higher plants where galactinol synthase (GolS; EC: 2.4.1.123) catalyzes the key step in RFO biosynthesis. These RFOs participate in many physiological processes like translocation of photoassimilates, abiotic stress tolerance, seed desiccation tolerance etc.^1^,^2^,^3^,^4^. Apart from these functions, RFOs were recently shown to act as signaling molecules upon pathogen attack and wounding^5^,^6^,^7^,^8^. RFOs are generally non-structural, non-reducing but soluble oligosaccharides present at high concentrations within the cell. The RFO biosynthetic pathway is initiated by the synthesis of galactinol (1-O-α-D-galactopyranosyl-L *myo*-inositol) which subsequently serves as a galactosyl donor and provides galactose moieties to the sucrose for the synthesis of raffinose. Further sequential addition of galactosyl group to the chain leads to the generation of series of RFOs like stachyose, verbascose and ajugose^9^,^10^. Galactinol is synthesized from UDP galactose and *myo*-inositol by the action of GolS which is considered as the key regulatory enzyme of this pathway^11^. GolS is a member of glycosyltransferase 8 (GT8) family and is usually encoded by a small gene family in higher plants. In *Arabidopsis*, GolS enzymes are encoded by a family of seven distinct genes which are spatially and developmentally regulated^2^. Studies have also shown that disruption of *AtGolS1* gene resulted in a decrease in galactinol and raffinose content after heat stress^12^. *Arabidopsis* plants overexpressing *AtGolS2* exhibited improved tolerance to drought stress^2^. Differential transcriptional regulation of the members of the galactinol synthase gene family was also observed in several other plant species. An increase in the production of galactinol and RFOs, as a consequence of coordinated transcriptional induction of the *GolS* coding genes in response to various abiotic stresses has been reported in several plant species^13^,^14^,^15^,^16^,^17^. In addition, *ZmGolS2* was found to be target of ZmDREB2A transcription factor and ZmGolS offers fairly similar protection against abiotic stresses upon overexpression in *Arabidopsis* plant^18^. Furthermore, accumulation of galactinol and RFOs during late maturation stages in few plant species particularly in legumes suggests their potential role in seed desiccation tolerance and longevity in dry state^3^,^9^,^19^. Very recently, de Souza *et al*.^20^ reported that galactinol content of dry mature seeds can be used as a suitable bio marker for seed longevity.

However, this hypothesis is still a matter of controversy^21^,^22^ and requires more studies to understand the precise role of RFOs in seed desiccation tolerance and longevity. Like many other legumes, chickpea seeds are also known to accumulate high amount of RFOs^23^. However, no detailed study of galactinol synthase and RFOs has been carried out in chickpea so far. Further, inositol metabolism which is known to regulate galactinol and RFO biosynthesis^24^,^25^,^26^ was shown to be upregulated during dehydration stress and reported to play an important role in seed physiology, seedling growth and stress tolerance in chickpea^27^,^28^,^29^,^30^. Though ample studies have been done on inositol metabolism in chickpea, the role and regulation of galactinol synthase has not been studied in this plant species. Considering all these, we aim to characterize the role and regulation of galactinol and GolS enzymes in chickpea. Chickpea, being a rich source of proteins is considered as one of the major sources of human food for developing countries. Unfortunately, the productivity of this grain legume is usually low and further reduced by environmental stresses^31^. Furthermore, chickpea seeds are sensitive to aging and a major concern for seed storage particularly in humid tropical climate. Even though there is an enormous agronomic and nutritional importance, research in chickpea is rather restricted due to the lack of mutant resources and efficient and dependable transformation protocol.

In present study in chickpea, we report that galactinol synthase activity is differentially regulated in different organs. Further, galactinol synthase activity along with galactinol and raffinose content increase during seed maturation and seed aging. Subsequently, we identified and cloned two *CaGolS* genes (*CaGolS1* and *CaGolS2*) which are found to produce five different transcript variants. Accumulation of these *CaGolS* transcript variants have been analyzed in different organs, during seed development and germination through qRT-PCR. Biochemical analysis revealed that only two (CaGolS1 and CaGolS2) among five CaGolS isoforms are biochemically active. Further analysis also revealed that both these isoforms exhibit similar yet distinct biochemical properties. Subcellular localization of these GolS isoforms has also been determined. Finally implication of these isoforms in seed vigor and longevity has been investigated through seed specific overexpression in *Arabidopsis thaliana*.

## Results

### GolS activity and consequent galactinol and raffinose content are markedly enhanced during seed development in chickpea

**Figure 1:**
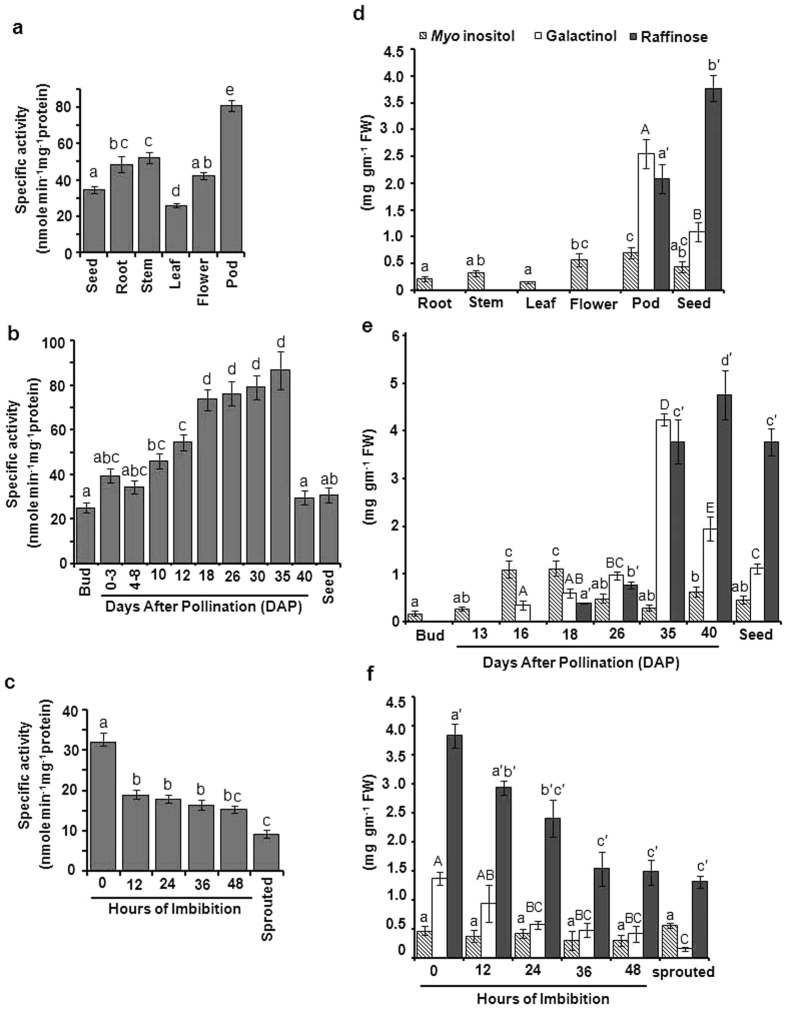
GolS activity profile (**a**–**c**) and Accumulation of galactinol, raffinose and inositol (**d**–**f**) in chickpea. GolS activity was determined: **(a)** in different organs; **(b)** during seed development; **(c)** during seed germination. Fifty μg of crude protein was used for the assay (sprouted indicates germinated seeds after 60 hrs imbibition). Specific activity was calculated nanomole Pi (inorganic phosphate) released per mg of protein per min. Data are means ± SD of four biological repeats. Significant differences among means (α = 0.01) are denoted by the different letters. Accumulation of galactinol, raffinose and *myo*-inositol **(d)** in different organs; **(e)** during seed development; **(f)** during seed germination. Polar metabolites were isolated, derivatized then metabolite content was quantified through GC-FID analysis. Data are means ± SD of four biological repeats. Significant differences among means (α = 0.01) are denoted by the different letters (*myo*-inositol in small letter, galactinol in capital letter and raffinose in small letter with prime symbol).

In order to explore the role and regulation of galactinol synthase in chickpea, initially different tissues were analyzed for the galactinol synthase activity. For this, total protein was extracted from different organs and was assayed as described in Methods section. As shown in Fig. 1a, differential galactinol synthase activity was observed in different tissues. The maximum level of GolS activity was detected in pod followed by stem, root, flower and seed (Fig. 1a). Further to obtain more detailed picture of galactinol synthase activity profile during the course of seed development in chickpea, flowers were tagged according to the day after pollination (DAP) (Supplementary Fig. S1) and subsequently total protein was extracted and activity was measured. As shown in Fig. 1b, GolS activity gradually increased as seed development proceeds and then maximum activity was observed at 35 DAP. However the activity sharply decreased at 40 DAP and in dry seeds. The activity was also monitored during the course of germination and data revealed that GolS activity gradually decreased during the course of germination and further reduced in germinated seed (Fig. 1c). Simultaneously, galactinol along with *myo*-inositol and raffinose content was also quantified in different organs, during seed development and germination. Galactinol accumulation was found to be maximum in pod followed by seed but undetectable in other organs. Raffinose was also undetectable in other organs except pod and seed. However, unlike galactinol accumulation, maximum level of raffinose accumulation was observed in seeds (Fig. 1d). Even though we have observed significant enzyme activity in vegetative organs in chickpea but galactinol and raffinose accumulation are not detectable in this organs possibly GC-FID is not sensitive enough to detect below certain level of these metabolites. As expected, *myo*-inositol was detected in all organs with slightly higher level in flowers and pods (Fig. 1d). During the early phase of seed development both galactinol and raffinose were undetectable, however became detectable during the mid phase of seed development and then gradually increased till 35 DAP. Interestingly, galactinol content declined after 35 DAP while raffinose content continued to increase till 40DAP and became highly abundant in dry seeds. *Myo*-inositol content was observed throughout seed development (Fig. 1e). Similar to GolS activity; galactinol and raffinose content were reduced upon imbibition during the course of germination (Fig. 1f).

### GolS activity, galactinol and raffinose content is enhanced during artificial aging in chickpea

**Figure 2:**
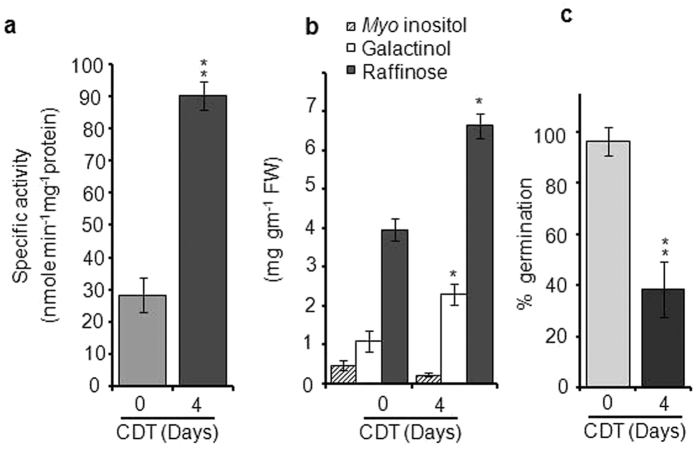
GolS activity (**a**) and accumulation of galactinol, raffinose and *myo*-inositol (**b**) and germination (%) (**c**) before and after CDT (4 Days) in chickpea. Seeds were imbibed to increase moisture content and (moisture content 22 ± 2%) were treated at 45 °C and 75% RH for 4 days to impose aging. For each biochemical assay 50 μg of crude protein was used and for each GC-FID analysis, polar metabolites were extracted from 300 mg of seed sample. Single and double asterisks indicate significant difference at P < 0.05 and P < 0.01, respectively.

Even though the galactinol synthase activity, galactinol and others RFO content have been analyzed in dry seed, during seed development and germination in a few plant species, such analyses have not been carried out in detail during the aging of seeds. Therefore to investigate this and towards exploring the potential role of galactinol and galactinol synthase in seed longevity, galactinol synthase activity along with galactinol, *myo*-inositol and raffinose were quantified after artificial aging. For this, seeds were subjected to Control Deterioration Test (CDT) and subsequently, enzyme activity, *myo*-inositol, galactinol and raffinose content were quantified. Results clearly revealed that galactinol synthase activity was significantly upregulated (*P* = *0.004*) after CDT (Fig. 2a). Likewise, galactinol and raffinose content significantly increased (*P* = *0.00393*) after CDT though *myo*-inositol level was found to be slightly reduced (Fig. 2b). However, seeds subjected to CDT showed a significant reduction (*P* = *0.000326*) in germination percentage (Fig. 2c). These analyses suggest that galactinol synthase(s) are upregulated during the aging of seeds and thereby accumulate increased level of galactinol and raffinose content in seeds, indicating the potential role of galactinol and raffinose in seed vigor and longevity.

### GolS is encoded by two divergent genes (*CaGolS1* and *CaGolS2*) in chickpea

**Figure 3:**
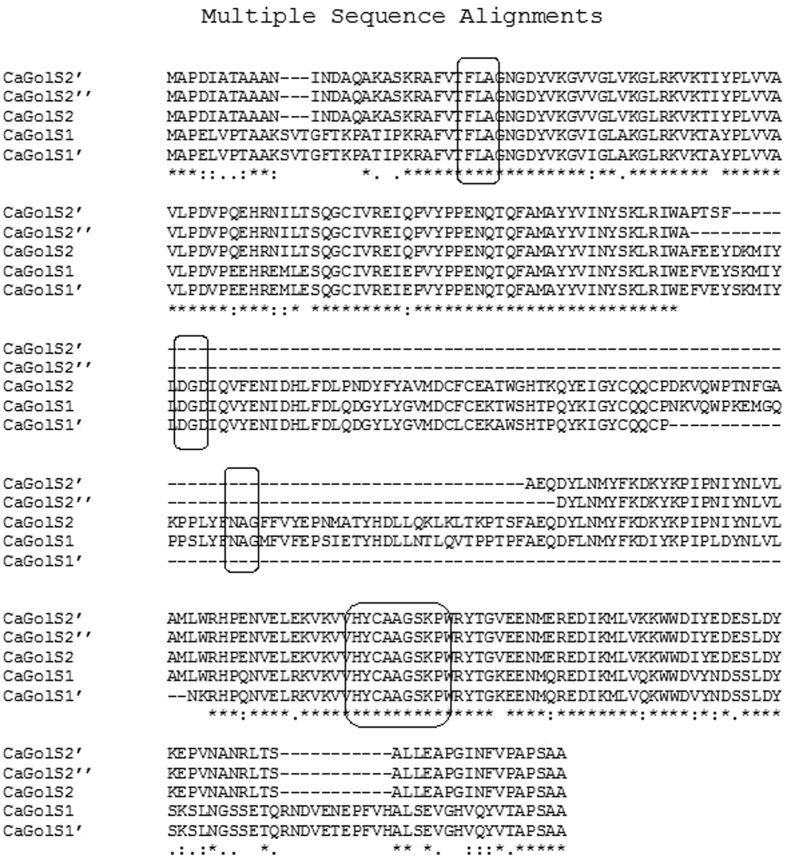
Multiple alignment of GolS protein sequences of chickpea using Clustal W 2.1 multiple alignment program. The GT8 family specific conserved DxD and HxxGxxKPW sequence motifs are highlighted in box. Galactinol synthase specific and putative active site domains are also highlighted in box (FLAG, DxD, NAG and APSAA).

GolS enzymes are encoded by a variable numbers of genes in different plant species. In order to identify galactinol synthase gene(s) in chickpea, we surveyed the chickpea genome sequence and two galactinol synthase coding genes were identified on chromosome 3 (*CaGolS1*) and chromosome 4 (*CaGolS2*). Subsequently, using specific primers, full length cDNA sequences of *CaGolS1* (Accession no: KU189226) and *CaGolS2* (Accession no: KU214571) were isolated and cloned. Sequence analysis revealed that *CaGolS1* contains an open reading frame of 1020 bp encoding 339 aa protein while *CaGolS2* contains an open reading frame of 978 bp encoding 325 aa protein. Like other GolS proteins, both CaGolS1 and CaGolS2 possess common features including DxD, HxxGxxKPW motifs and conserved sequences like NAG, FLAG. (Figure 3, Supplementary Fig. S2). DxD, NAG, FLAG amino acid residues are predicted to be important for GolS enzyme activity and further DxD motif is also shown to be required for divalent cation binding^32^.

Apart from *CaGolS1* and *CaGolS2*, we have identified and cloned three additional transcript variants (*CaGolS1*′, *CaGolS2*′ and *CaGolS2*″). *CaGolS1*′ (Accession no: KU189227) is 801 bp in length and potentially encode a 266 aa protein. The CaGolS1 isoform possess DxD, HxxGxxKPW motifs but lacks NAG sequence. *CaGolS2*′ (Accession no: KU214572) is 678 bp in length and encodes 228 aa protein while *CaGolS2*″ (Accession no: KU214573) is 657 bp in length and encodes 218 aa protein. Both these isoforms contain HxxGxxKPW motif but lack NAG and DxD motif. Upon comparison with genomic DNA sequences, transcript variants appear to be the result of alternative splicing of respective *CaGolS* genes (Fig. 3, Supplementary Fig. S2).

### *CaGolS1* and *CaGolS2* are differentially regulated in chickpea

**Figure 4:**
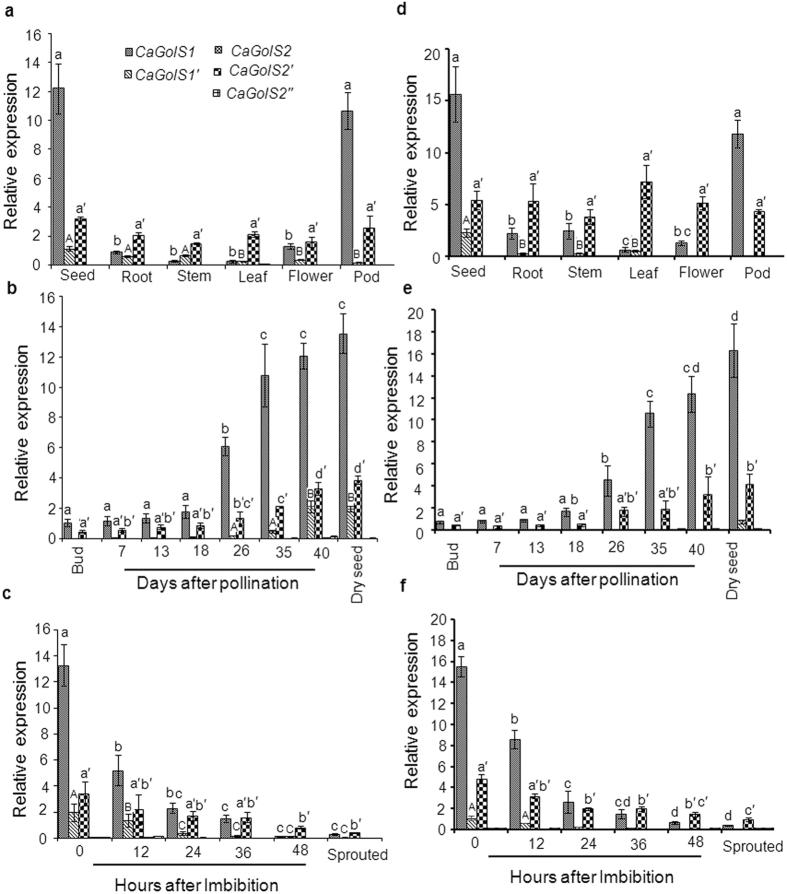
Quantitative RT PCR analysis of *CaGolS*(s) in (**a**,**d**) in different organs; (**b**,**e**) during seed development; (**c**,**f**) during seed germination. Total RNA from each sample was reverse transcribed and subjected to real time PCR analysis. The relative expression value of each gene was normalized using two endogenous control *18S* (**a**–**c**) and *EF1α* (**d**–**f**) and calculated using the ΔΔCT method (Applied Biosystems). Values are the result of triplicate analysis of three biological replicates. Error bars indicate the standard deviation. Significant differences among means (α = 0.01) are denoted by the different letters (*CaGolS1* in small letter, *CaGolS1*′ in capital letter and *CaGolS2* in small letter with prime symbol).

Our previous analysis showed distinct pattern of RFO accumulation and GolS activity in different organs, during seed development and germination in chickpea. This differential activity is apparently controlled by the precise expression of individual GolS coding gene(s). Therefore to understand the regulation of expression of *GolS1* and *GolS2* gene, accumulation of their transcripts (*CaGolS1, CaGolS1*′, *CaGolS2, CaGolS2*′ and *CaGolS2*″) has been studied through qRT-PCR. For this purpose, we used two different endogenous controls (*EF1α* and *18S rRNA*) for normalization yet yielded similar results. Results revealed that *CaGolS1* and *CaGolS2* genes were found to be differentially expressed in different organs and all the transcript variants were accumulated maximum in seeds (Fig. 4a,d). *CaGolS1* transcript accumulation was found to be more in reproductive tissues than *CaGolS2* transcript, while in vegetative tissues, accumulation of *CaGolS2* transcript was slightly greater as compared to *CaGolS1* (Fig. 4a,d). Other transcript variants (*CaGolS1*′*, CaGolS2*′ and *CaGolS2*″) were found to be accumulated in very low levels in all organs. Accumulation of these transcripts was also analyzed during the course of seed development and germination. As shown in Fig. 4b,e, both *CaGolS1* and *CaGolS2* transcripts were found to be progressively increased as the seed development proceeded; though the *CaGolS1* was found to be more induced than *CaGolS2* during seed development. Transcript accumulation for both *CaGolS1* and *CaGolS2* was found to be gradually decreased as seed germination proceeds and was reduced further after the completion of germination (Fig. 4c,f). A very low level of transcripts was observed in case of other variants (*CaGolS1*′*, CaGolS2*′ and *CaGolS2*″) during seed development and germination.

### CaGolS1 and CaGolS2 exhibit similar yet distinct biochemical properties

**Figure 5:**
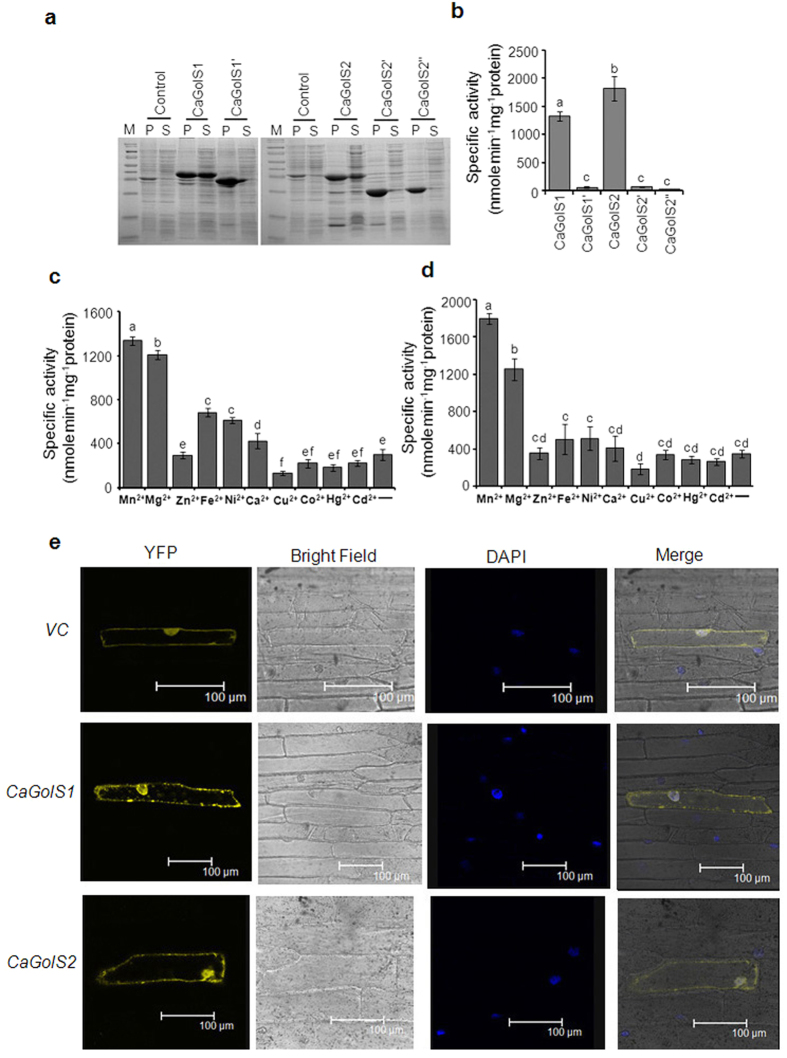
Biochemical characterization (**a**–**d**) and sub-cellular localization (**e**) of CaGolS isoforms. **(a)** 12% SDS PAGE analysis of CaGolS(s) over expressed protein in *E. coli* Bl21 (DE3). [Control- pET23b empty vector transformed induced cells; M- molecular weight marker; P- Pellet fractions; S- Soluble fractions]. **(b)** Comparison of enzyme activity among CaGolS1, CaGolS1′, CaGolS2, CaGolS2′ and CaGolS 2″. Five μg purified protein was assayed in each case. **(c,d)** Effect of divalent cations on purified recombinant CaGolS1 and CaGolS2. CaGolS activity was assessed in presence of various divalent cations (4 mM). Error bars indicate the deviation from three independent experiments. Significant differences among means (α = 0.01) are denoted by the different letters. **(e)** Subcellular localization of CaGolS1 and CaGolS2. The CaGolS–YFP fusion construct and the YFP control plasmid were introduced into the onion peel epidermal cells by particle bombardment. Expression of the introduced genes was examined after 48 h by confocal microscopy. Nuclei were stained by 4′, 6-diamino-phenylindole (DAPI), Column1 shows YFP fluorescence, column 2 shows onion peel cells imaged under bright field, column 3 shows DAPI staining and column 4 shows merge of bright field, fluorescence and DAPI. Scale bar represents 100 μm.

Since we observed considerable differences in amino acid sequences among CaGolS isoforms, we were interested to examine the catalytic activity and enzymatic properties of these CaGolS isoforms. Furthermore, the absence of important motifs like DxD and NAG in alternative splice variants (CaGolS1′, CaGolS2′ and CaGolS2″) also prompted us to examine whether these isoforms were able to synthesize galactinol from UDP galactose and *myo*-inositol in *invitro* condition. Therefore, respective cDNAs were cloned into bacterial expression vector pET-23b and were heterologously expressed as a hexa his-tagged recombinant protein in *E. coli* BL21DE3. CaGolS1 and CaGolS2 were expressed in both soluble and particulate fraction while CaGolS1′, CaGolS2′ and CaGolS2″ were expressed predominantly in particulate fraction (Fig. 5a). All isoforms were purified from respective fractions through affinity chromatography using His Trap Ni-NTA columns as described in Methods section. Purified fractions were then used to determine the enzyme activity and biochemical properties. As shown in Fig. 5b, only CaGolS1 and CaGolS2 exhibited enzyme activity while all other splice variants (CaGolS1′, CaGolS2′ and CaGolS2″) showed barely any considerable activity. Further, enzymatic properties such as temperature optimum, pH optimum, *Vmax* and *Km* values for *myo*-inositol and UDP galactose were also determined for CaGolS1 and CaGolS2. Both the recombinant enzymes showed similar pH optimum of 7.5 however exhibited different temperature optimum. CaGolS1 had temperature optimum at 40 °C while CaGolS2 exhibited maximum activity at 30 °C (Table 1). The apparent *Km* and *Vmax* values for UDP-galactose of CaGolS1 and CaGolS2 were somewhat comparable to each other. However interestingly, CaGolS1 and CaGolS2 isoforms differed in respect to their *Km* and *Vmax* values for *myo*-inositol. CaGolS2 apparently had higher *Km* and *Vmax* values in respect to *myo*-inositol. Results are summarized in Table 1. Further, requirement of Mn^2+^ for the catalytic activity of these two enzymes was checked. In absence of MnCl_2_, both enzymes showed bare minimum activity while addition of 4 mM MnCl_2_ in reaction mixture provided maximum activity. In addition to Mn^2+^, few others divalent cation (Mg^2+^, Fe^2+^) also observed to enhance the enzyme activity. As opposed to this, few cations such as Co^2+^, Cu^2+^, and Hg^2+^ happened to reduce the catalytic activity of both these enzymes (Fig. 5c,d).

Further, native molecular mass of these CaGolS isoforms was determined through size exclusion chromatography using Sephacryl S200HR column as described in Methods section. Both CaGolS1 and CaGolS2 were eluted in fractions corresponding to region with apparent molecular weight of 35–45 kDa, suggesting the monomeric nature of these proteins (Supplementary Fig. S3).

### CaGolS1 and CaGolS2 exhibit similar subcellular localization

Despite the expression pattern of galactinol synthase being documented in several studies, their subcellular localization has not been properly investigated, though some indirect evidences indicate there to be cytosolic^33^,^34^. However, according to some recent reports, GolS localize to the cell membrane^35^ and nucleus^36^. Therefore, to examine the subcellular localization of CaGolS1 and CaGolS2, respective cDNAs were cloned under the control of 35S promoter and expressed transiently as N-terminal YFP fused protein in onion peel epidermal cell. As shown in Fig. 5e, YFP fused GolS proteins were observed predominantly in the nucleus and plasma membrane and also detectable in cytosol. Localization of nucleus was confirmed by staining with DAPI dye.

### Seed specific over expression of *CaGolS1* and *CaGolS2* results in enhanced galactinol and raffinose content and improves seed germination vigor, longevity in *Arabidopsis thaliana*

In our earlier experiments, we observed upregulated galactinol synthase activity with consequently increased accumulation of galactinol and raffinose content during maturation drying. Further, artificial aging also stimulated galactinol synthase enzyme activity and enhancement of galactinol and raffinose content in seed. Hence, we were interested to evaluate the potential role of galactinol synthase in seed vigor and longevity. Therefore, *CaGolS1* and *CaGolS2* were expressed in seed specific manner in *Arabidopsis* under the control of napin promoter. *CaGolS1* and *CaGolS2* transformed plants were initially selected by kanamycin resistance followed by *GUS* reporter gene expression. Further, the presence of *CaGolS1* and *CaGolS2* in respective transgenic lines was confirmed by the PCR analysis using specific primers. The transgenic lines exhibiting 3 kan^R^: 1 kan^S^ segregation pattern in its progeny were selected and subsequently homozygous lines for *CaGolS1* and *CaGolS2* were used for functional analysis. Transcript expressions of the transgene (s) were checked in several independent lines and a greater transcript accumulation of *CaGolS1* and *CaGolS2* were observed in respective transgenic lines (Supplementary Fig. S4). Overall higher total GolS activity in seed was also observed in transgenic lines (Supplementary Fig. S5).

**Figure 6:**
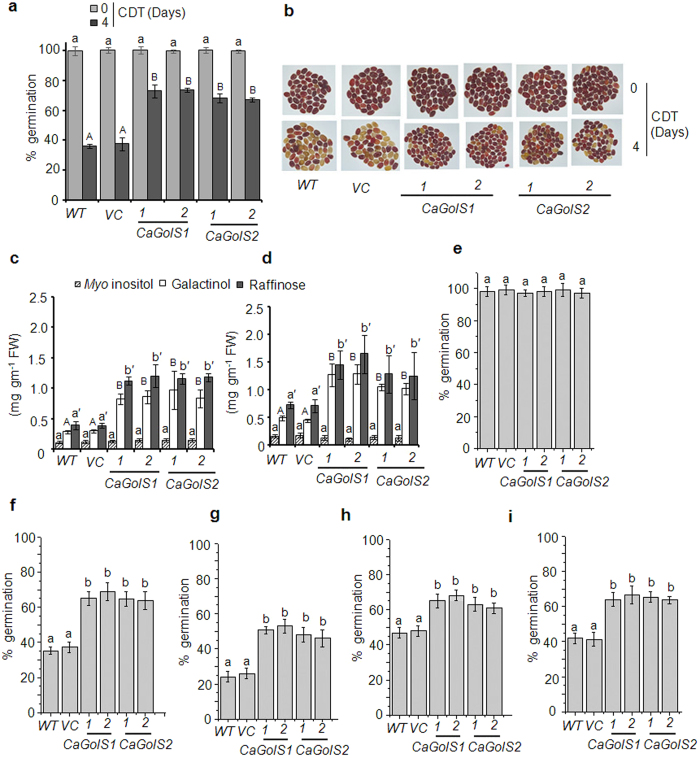
*CaGolS1* and *CaGolS2* improves seed vigor and longevity. Experiments on two representative independent transformed lines (For *CaGolS1* 1: L2, 2: L4; For *CaGolS2*1:L1, 2: L5) of each gene are shown here. Eight week old seeds were imbibed to increase moisture content (24% ± 2) and then subjected to CDT (45 °C and 75% RH) for 0 to 4 days. Germination was scored after 7 days of imbibition. **(a)** Germination percentage of wild type (*WT*), empty vector (*VC*), *CaGolS1*, and *CaGolS2* transformed *Arabidopsis* seeds before and after CDT. Data are means ± SD of three biological repetitions with 50 seeds each. **(b)** Viability of seeds in wild type, vector control, and respective transformed lines after CDT (0 to 4 days). Seed viability was analyzed using tetrazolium staining and dark red staining indicates seeds are viable. **(c,d)** Quantitation of galactinol, raffinose and *myo*-inositol accumulation in seeds of wild type (*WT*), vector control (*VC*), *CaGolS1*, and *CaGolS2* transformed lines **(c)** before and **(d)** after CDT. For GC-FID analysis, polar metabolites were extracted from 50 mg of seeds. Data are means ± SD of three biological repeats. **(e–i)** Comparison of germination performance among seeds of wild type (*WT*), empty vector (*VC*), *CaGolS1*, and *CaGolS2* transformed lines under various stress conditions. Dry mature seeds from all genotypes were germinated under various stress conditions. [E-control, F-Heat (45 °C), G-Paraquat (1 μM), H-Sodium chloride (200 mM), I-PEG (−0.5 MPa)]. Data are means ± SD of four repetitions with 50 seeds each. Significant differences among means (α = 0.01) are denoted by the different letters. [For C and D figures, *myo*-inositol in small letter, galactinol in capital letter and raffinose in small letter with prime symbol).

Subsequently, we have examined the potential improvement of seed vigor and longevity of *CaGolS1* and *CaGolS2* overexpressing *Arabidopsis* seeds. To asses this, we have conducted CDT as described in Method section and then germination and viability of these seeds were evaluated. Under normal conditions, *CaGolS1* and *CaGolS2* transformed seeds as well as control (wild type and vector control) seeds showed no differences with respect to germination percentage (100% germination). However, after 4 days of CDT, *CaGolS1* and *CaGolS2* transformed seeds exhibited strikingly 65 to 73% germination while only 35-38% control seeds (wild type and vector control) germinated (Fig. 6a, Supplementary Fig. S6). Further to examine whether this improved seed germination of *CaGolS* transgenic lines were correlated with seed viability in transgenic lines, tetrazolium (TZ) assay was performed^37^. *CaGolS1*, *CaGolS2*, and control seeds were evenly stained a dark red color before subjected to CDT. However after CDT, only *CaGolS1* and *CaGolS2* transformed seeds showed dark red staining in contrast to control seeds which remained unstained or stained pale red (Fig. 6b). This results clearly indicated that *CaGolS* transformed seeds were viable even after aging treatment. Next, we examined whether this improved seed germination after aging is associated with increased galactinol and raffinose accumulation in seeds of *CaGolS1* and *CaGolS2* transgenic lines, we quantified galactinol and raffinose content in these seeds before and after CDT. Results showed that galactinol and raffinose content were significantly higher in transgenic lines as compared to control lines both before and after CDT (Fig. 6c,d). Further, we have also examined the influence of raffinose on germination of the wild type *Arabidopsis* seeds after CDT. Results showed that after CDT germination was less inhibited when supplemented with raffinose (Supplementary Fig. S7). To check whether over expression of galactinol synthase in seed can enhance seed germination vigor, their germination ability against various environmental stresses such as heat, oxidative, salinity and dehydration stress was tested. Results showed that seeds from both *CaGolS1* and *CaGolS2* overexpression lines exhibited improved seed germination compared to control lines in all stress conditions tested (Fig. 6e–i).

### Improved seed vigor and longevity of *CaGolS1* and *CaGolS2* overexpressing lines are associated with reduced Reactive Oxygen Species (ROS) accumulation and MDA content

**Figure 7:**
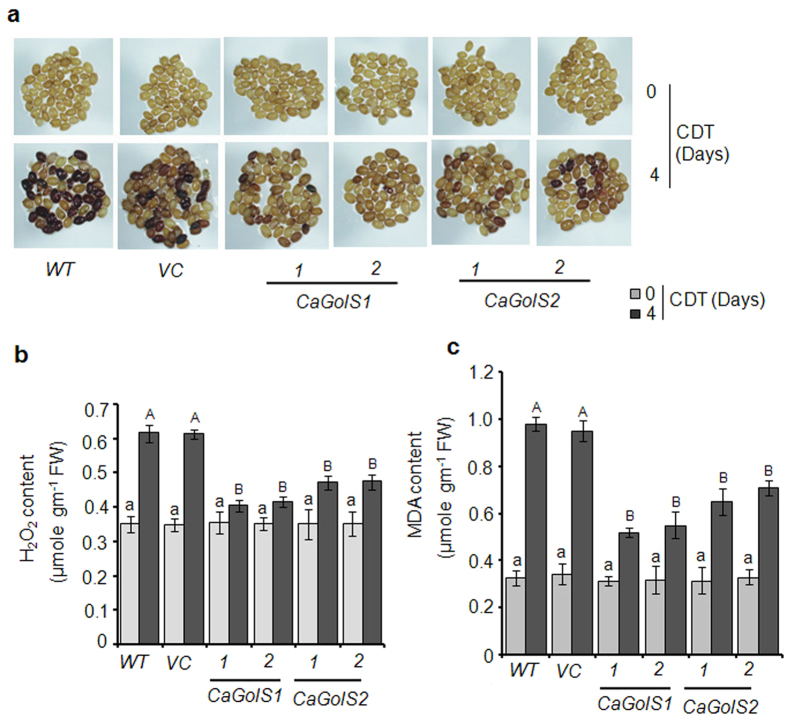
*CaGolS1* and *CaGolS2* lines exhibit reduced ROS accumulation. Experiments on two representative independent transformed lines (For *CaGolS1* 1: L2, 2: L4; For *CaGolS2*1:L1, 2: L5) of each gene are shown here. **(a)** Comparison of H_2_O_2_ accumulation among seeds of wild type (*WT*), empty vector (*VC*), *CaGolS1* and *CaGolS2* lines before and after CDT (4 days). Dark staining of seed indicates H_2_O_2_ accumulation. **(b,c)** Quantitative analysis of **(b)** H_2_O_2_ and **(c)** MDA content in seeds of wild type (*WT*), vector control (*VC*), *CaGolS1* and *CaGolS2* transformed lines before and after **(b,c)** CDT. Data are means ± SD of three biological repeats. Significant differences among means (α = 0.01) are denoted by the different letters.

Restriction of ROS accumulation during seed aging is one of the important mechanisms for maintaining seed vigor and longevity as increased ROS accumulation and subsequent lipid peroxidation, protein damage have been reported with progressive seed aging^38^. Interestingly, galactinol has recently been reported to act as antioxidative molecule and has the ability to scavenge hydroxyl radicals^14^. Therefore to examine whether improved seed vigor and longevity is associated with reduced ROS accumulation in transgenic lines, we initially checked the H_2_O_2_ content through DAB staining. Results showed that H_2_O_2_ content in seeds increased after CDT treatments in all genotypes. However strikingly, *CaGolS* over expressing lines exhibited light brown staining indicating less accumulation of H_2_O_2_ than control lines which accumulated more H_2_O_2_ and thus stained dark brown (Fig. 7a). These data was further confirmed by the quantitative analysis of H_2_O_2_ in these transgenic and control lines and similar results were observed (Fig. 7b). Further to examine whether reduced ROS content of *CaGolS* transgenic lines result in reduced lipid peroxidation, MDA content in transgenic seeds and control seeds (wild type and vector control) before and after CDT was also analyzed. As expected, MDA content was accumulated in lesser extent in *CaGolS* overexpressing transgenic seeds as compared to control seeds particularly after CDT (Fig. 7c). These results clearly suggest that *CaGolS* overexpressing seeds exhibited reduced ROS mediated cellular damage than control seeds.

Altogether our data strongly demonstrated that *CaGolS* transformed lines accumulate increased galactinol and raffinose content and exhibit improved seed vigor and longevity by limiting age induced excess ROS accumulation in seed.

## Discussion

Increased accumulation of RFOs during seed maturation has been observed in several plant species and is suggested to play an important role in the acquisition of seed desiccation tolerance and consequent seed longevity^39^,^40^. However, the role of RFOs in such seed traits still remain elusive and a matter of controversy. Few studies showed that large amount of RFOs are not really essential for seed desiccation tolerance or seed longevity^21^,^22^. In the present study, we clearly demonstrated that galactinol synthase, the rate limiting enzyme of RFO biosynthesis, plays an important role in seed vigor and longevity. We showed that galactinol synthase activity progressively increases till late maturation phase during seed development of chickpea however declines when the seed reaches at the very late stage of maturity. GolS activity reduces further following imbibition during germination. Likewise, galactinol accumulation reaches maximum at 35DAP and then declines subsequently. Similar observations were also reported in few other legumes including *Glycine max, Vicia hirsuta*, and thus support their participation in seed desiccation tolerance and longevity^19^,^41^,^42^. Like many other species, raffinose content also reaches maximum level at the very late stage of maturity and becomes highly abundant in dry chickpea seeds. The pattern of GolS activity along with galactinol and raffinose accumulation also reflects that galactinol is mostly utilized for raffinose synthesis during the very late stage of seed maturation, as galactinol content reduces and raffinose content increases towards the end of seed maturation. Both galactinol and raffinose content begin to decline following imbibition. Previous reports clearly demonstrated that a range of protective molecules which are generally associated with desiccation tolerance and longevity are highly accumulated during maturation phase of orthodox seeds and then decline following germination^38^,^43^,^44^,^45^,^46^,^47^. Therefore upregulation of galactinol synthase activity and consequent increased accumulation of galactinol and raffinose during seed maturation in chickpea also indicates their participation in seed desiccation tolerance and seed longevity. Furthermore, artificial aging induced stimulation of galactinol synthase activity and resulting increased accumulation of galactinol and raffinose in chickpea seed also strengthens our hypothesis that galactinol synthase and RFOs indeed participate in seed desiccation tolerance and longevity in chickpea. Like many other species^48^,^49^, galactinol synthase enzymes in chickpea are encoded by two divergent genes (*CaGolS1* and *CaGolS2*). However, chickpea *GolS* genes produce various transcript variants that potentially encode several CaGolS isoforms. Among them, only two isoforms (CaGolS1 and CaGolS2) are found to be biochemically active. While three isoforms (CaGolS1′, CaGolS2′ and CaGolS2″) are rather biochemically inactive and lack of such biochemical activity could be due to the deletion of the important motifs such as NAG, DxD etc. in their protein sequences^32^. However the presence of such enzymatically inactive isoforms in chickpea is at present unclear. Interestingly, CaGolS1 and CaGolS2 isoforms exhibit distinct yet similar biochemical properties. CaGolS1 showed maximum activity at moderately high temperature. Maintaining high GolS activity to synthesize galactinol and subsequent RFOs in harsh conditions may particularly be important in maintaining seed desiccation, vigor and longevity. Several studies also suggest that seed desiccation tolerance and longevity associated proteins are often heat stable and are induced at high temperature^50^. Further our expression analysis also suggests that *CaGolS1* is predominantly expressed in developing and mature seeds and thus likely to play a key role in seed desiccation tolerance and longevity. While CaGolS2 possibly play major role in synthesizing galactinol in vegetative tissues as *CaGolS2* transcript level is comparatively greater than *CaGolS1* in vegetative tissues and are less influenced by seed developmental process. Similar pattern of expression was also observed in few other species including, *Pisum sativum, Lycopersicon esculentum* and *Vicia hirsute* where *GolS1* gene was observed to be induced during seed development and suggests their potential role in seed desiccation tolerance^3^,^13^,^42^. Considering the previous reports and our expression pattern and biochemical properties, it seems that *CaGolS1* evolved to play a major role in seed desiccation tolerance while *CaGolS2* is important for other physiological roles in chickpea. Subsequent analysis also showed that seed specific expression of *CaGolS1* and *CaGolS2* results in improved seed vigor and longevity in transgenic *Arabidopsis*. Further, our results also demonstrated that seed specific expression of galactinol synthase not only results in the enhancement of galactinol content but also enhances raffinose content in seed. Our results clearly indicated the positive correlation of improved seed vigor and longevity with increased accumulation of galactinol and raffinose content. Interestingly, *CaGolS1* lines exhibit slightly higher galactinol and raffinose content than *CaGolS2* transgenic lines particularly after CDT, possibly because CaGolS1 enzyme has the capability to synthesize galactinol even at higher temperature. Though, further studies will be required to validate that increased GolS activity leads to improved storage behavior at more ambient temperature through natural aging. Previous study revealed that, galactinol and raffinose have the ability to scavenge hydroxyl radicals and play important role in protection against oxidative stress in plants^14^. Importantly, during seed maturation, desiccation, dormancy to the early phase of germination, seed, particularly the embryo, faces severe dehydration and oxidative stress that can potentially leads to excess ROS accumulation^51^,^52^,^53^,^54^. This excess accumulation of ROS during seed storage is one of the main causes for seed deterioration and reduced seed vigor and longevity^55^. Therefore restricting ROS level associated with cellular damage is one of the important mechanisms to maintain seed vigor and longevity in orthodox seeds. In our study, we observed that improved seed vigor and longevity of *CaGolS* transgenic lines are associated with reduced level of ROS and consequently reduced lipid peroxidation.

Collectively, our results strongly suggest that galactinol synthase are differentially regulated in chickpea to play an important role in maintaining seed vigor and longevity by restricting excess ROS and consequent cellular damage.

## Methods

### Plant material and growth condition

*Cicer arietinum* L. cv BGD72 was used in this study. Chickpea seeds were germinated and grown as described previously^27^,^30^. *Arabidopsis thaliana* Col-0 ecotype was used for transformation. Wild type and all transgenic lines were grown in plant growth facility centre (Conviron) maintained at 22 °C ± 2 °C with 16/8 hrs light (200 μmol m^−2^ sec^−1^)/dark cycle for general growth and seed harvesting. Seeds (from mature brown silique) were harvested on the same day from all plants and then stored in the dark under dry condition at room temperature (24 °C ± 2 °C) for at least 8 weeks before used for experiments. Germination frequency or other parameters were evaluated in four replicates using 50 seeds.

### Isolation and molecular cloning of the *GolS* cDNAs from chickpea

Total RNA was isolated from chickpea seedlings using TRI reagent (Sigma) and then cDNA was prepared using superscript III reverse transcriptase (Invitrogen). Gene specific primers were designed based on sequence information available in chickpea genome sequence and transcriptome database (CTB) (http://www.nipgr.res.in/ctdb.html) and were used to amplify full length cDNA sequences of *CaGolS*(s). The amplified PCR products were cloned into pJET1.2 vector (Thermo scientific) and subsequently sequenced. All primer sequences are provided in Table S1.

### Quantitative real-time PCR

Total RNA was isolated from vegetative organs and flower using TRI reagent (Sigma) following manufacturer’s instruction while from dry and imbibed seeds, RNA was extracted according to Singh *et al*.^56^. 2 μg of total RNA was reverse transcribed using random primers (using ABI cDNA synthesis kit). Real-time PCR reactions were run on an ABI Step one real time PCR using specific primer pairs for *CaGolS* transcripts and an endogenous control *18S rRNA* and elongation factor 1-alpha (*EF1α*)^57^. A negative control lacking cDNA sample was also included in each assay. All reactions were performed in triplicate with three biological replicates. All primer sequences are provided in Table S1.

### Protein extraction and GolS assay

Tissue sample (0.5 g) was finely ground in liquid N_2_ using mortar and pestle and then homogenized with extraction buffer (100 mM HEPES [4-(2-hydroxyethyl)-1-piperazineethane sulfonic acid] pH 7.5, 1 mM β mercapto ethanol, and protease inhibitor cocktail [Sigma])^14^,^58^. The homogenate was spun at 10000 g for 10 min and the supernatant was collected and used for the assay or stored at −80 °C till further use. The protein content of these soluble extracts was estimated using Bradford^59^ method using BIO-RAD protein estimation kit. For GolS enzyme assay, a colorimetric assay was performed as described by Ribeiro *et al*.^60^. In brief, reaction mix [60 mM *myo*-inositol, 2 mM DTT, 50 mM HEPES buffer (pH 7.0), 4 mM MnCl_2_, 20 μg of bovine serum albumin and 4 mM UDP-gal and crude (50 μg) or purified protein (5 μg)] were incubated at 32 °C for 1 h and then in boiling water for 2 min to stop the reaction. Apyrase reaction mixture with potato apyrase (0.3 U) was added to the assay reaction mix and was incubated at 37 °C for 10 min. Further 75% of TCA was added to the reaction mix and incubated in ice for 10 min. Reaction mix was centrifuged at 3000 g for 10 min and supernatant was used to estimate inorganic phosphate by Fiske subbarow protocol^61^.

### Bacterial over expression and purification of recombinant CaGolSs

In order to characterize the CaGolSs, respective cDNAs were sub cloned into the NdeI/XhoI sites of the bacterial expression vector pET23b (Novagen) and subsequently proteins were expressed with a C-terminal histidine tag in *E. coli* BL-21(DE3) cells. Transformed cells were induced by using 0.5 mM IPTG and then allowed to express for 8 h at 25 °C. The expressed protein which was found in particulate fraction was solubilized in 8 M urea buffer and then purified as described by Majee *et al*.^62^. Protein present in soluble fraction was also purified similarly using nickel charged affinity columns (GE Healthcare) following the manufacture’s protocol.

### Gas Chromatography –Flame Ionization Detector (GC-FID)

All solvents (methanol, water and ethanol), sugar standards (*myo*-inositol, galactinol and raffinose) and derivatization reagent methoxyl amine hydrochloride (CH_5_NO.HCl), pyridine (C_5_H_5_N), N-methyl-N-(trimethylsilyl) trifluoroacetamide (MSTFA) used were of GC grade (Sigma).

Polar metabolites were isolated according to Panikulangara *et al*.^12^ and completely dried in lyophilizer. Subsequently samples were derivatized according to the method described by Agarwal *et al*.^63^. Derivatized samples were then centrifuged at 14,000 g for 5 min and transferred to fresh glass vial. The GC analysis was carried out on a Shimadzu GC-2010 system equipped with flame ionization detector (FID). Phenomenex Rxi-1ms GC column (Restek Corporation) with 0.25 μm thickness, 30 m length and 0.25 mm diameter was used with nitrogen as carrier gas (N_2_). The injector temperature was maintained at 300 °C. The program of column temperature was set at 230 °C during the injection of sample and then holds for 1 min. Subsequently temperature was to increase at 7 °C min^−1^ to reach 260 °C, and kept constant at 260 °C for 1 min and then temperature again increased to 290 °C at a rate of 3 °C min^−1^ followed by an isothermal hold at 290 °C for 13 min. Standard solutions of soluble carbohydrates (*Myo*-inositol, Galactinol and Raffinose) of six different concentrations were also derivatized and analyzed by GC–FID. Standard graph for each was plotted with known concentration of standards and linear equations were defined. The amount of sugars present in the sample was calculated based on computer integration of the peak areas in the GC chromatograms.

### Gel filtration Chromatography

To determine the native molecular weights of recombinant CaGolS(s) proteins, size exclusion chromatography was carried out on AKTA Prime Plus system using Sephadex 200HR column (GE). Column was equilibrated with two column volumes (240 ml) of running buffer containing 20 mM Tris-Cl buffer, pH 7.6, 150 mM NaCl, 2 mM β-ME and 10% glycerol and then sample was run at a constant flow rate of 0.4 ml min^−1^. Column was initially calibrated with known molecular weight standards (Sigma) and then Vo and Ve for each standard were determined.

### Vector construction and *Arabidopsis* transformation

To generate seed specific expression of *CaGolS*(s) in plants, full-length *CaGolS1* and *CaGolS2* CDS were sub cloned into pCAMBIA 2301 plant expression vector under the control of the napin promoter. Constructs were initially transformed to *Agrobacterium tumefaciens* strain GV3101 and then finally transformed to *Arabidopsis thaliana* by Floral Dip method^64^. Transformed lines were seclected against kanamycin resistance.

### Subcellular localization of CaGolS(s)

To study sub cellular localization, *CaGolS1* and *CaGolS2* were cloned in a N-terminal YFP fusion vector (pSITE:YFP3CA) using gateway technology. For this, entry clones of *CaGolS1* and *CaGolS2* were made in pENTR D TOPO vector using the Invitrogen gateway system according to the manufacturer’s instructions. Finally, constructs were generated in gateway destination vector pSITE:YFP3CA through entry clone using the Gateway LR Clonase II enzyme mix (Invitrogen). Constructs were confirmed by sequencing. The constructs were then transformed into onion epidermal cells using Biolistic PDS-1000/He Particle Delivery System (Bio-Rad). Fluorescence images were taken using laser confocal scanning microscope (Leica Microsystems).

### Control Deterioration Test (CDT)

CDT was performed as described in previous reports with some modification^37^,^65^,^66^. For both *Arabidopsis* and chickpea, CDT experiments were carried out at least in three biogical repeats in airtight container containing appropriate saturated solutions of NaCl to obtain stable 75% relative humidity. CDT has been carried out in seeds with incresed mositure content (24% ± 2). Seeds were placed in a suitable tube with lid opened in the sealed container maintained 75% RH and kept at 45 °C for 4 days. Treated seeds were then used for further analysis to evaluate viability, vigor, germination performance, RFO accumulation etc.

Seed moisture content was measured directly by loss or gain of seed weight after drying at 105 °C for 24 hrs according to standard method and formula. % moisture content = [Weight of seeds before drying−weight of seeds after drying]/[weight of seeds before drying] ×100.

### Germination assay

For seed germination assay, three biological repeats of 50 seeds each were analyzed. Seeds were surface sterilized with sodium hypochlorite and thoroughly washed with autoclaved MilliQ water and then kept in dark and cold (4 °C) for stratification for 2 days before kept at 22 °C ± 2 °C with 16/8 hrs light/dark cycle for 7 days. To evaluate germination under stress conditions sterilized seeds were plated on ½ MS medium supplemented with 200 mM NaCl for salt stress, 1 μM PQ for oxidative stress, −0.5 MPa PEG for dehydration. For heat stress, seeds were kept in water bath at 45 °C for 90 min followed by plating on ½ MS medium. Seed germination was considered to be completed when the radicle protruded beyond the testa and germination was scored after 7 days of plating.

### Tetrazolium assay

Tetrazolium assay was performed in three biological repeats with 50 seeds each as described by Verma *et al*.^37^ (http://www.bio-protocol.org/e884). Seeds were initially scarified with scarification solution [20% (v/v) commercial bleach with 0.1% (v/v) triton X100] and then rinsed with sterilized distilled water. Scarified seeds were then incubated in 1% tetrazolium solution in darkness at 30°C for 24h to stain. Viability of seeds is determined by the staining pattern and red color intensity of seed as tetrazolium precipitates to red colored 2, 3, 5 triphenyl formazan by the activity of dehydrogenases present in the live cells. Stained seeds were photographed using Zeiss SteREO Discovery V12 microscope fitted with Axiocam ICc3 camera.

### DAB staining

For H_2_O_2_ accumulation, DAB staining was performed according to the protocol described by Mao & Sun^67^ with minor modification. Scarified seeds were vacuum infiltrated with freshly prepared 2 mg ml^–1^ 3,3′-diaminobenzidine (DAB; Sigma-Aldrich) solution in 50 mM Tris acetate buffer (pH-3.8) and then incubated at 25 °C for 24 h in dark. Thereafter seeds were washed with 95% ethanol and were photographed using using Zeiss SteREO Discovery V12 microscope fitted with Axiocam ICc3 camera.

### H_2_O_2_ and MDA quantification

Hydrogen peroxide (H_2_O_2_) content was estimated by method described by Saxena *et al*.^30^. For this, 50 mg of *Arabidopsis* seeds were ground with 2 ml of 0.1% TCA and centrifuged at 13000 g for 20 min at 4 °C. The 0.5 ml of supernatant was used in a reaction mixture (0.5 ml 10 mM potassium phosphate buffer of pH 7 and 1 ml of 1 M potassium iodide) and incubated in dark for 1 h. Absorbance was measured at 390 nm. The H_2_O_2_ content was quantified using a standard curve plotted from known concentrations of H_2_O_2_.

MDA content was measured using method described by Heath & Packer^68^. In brief, 50 mg of seeds were homogenized with 2 ml of 0.25% thiobarbituric acid dissolved in 10% TCA and incubated at 95 °C for 30 min. Reaction mixture was kept on ice for 10 min followed by centrifugation at 13000 g for 30 min. The supernatant was used to measure absorbance at 532 nm and 600 nm. MDA concentration of samples was calculated by using extinction coefficient of 155 mM^−1^ cm^−1^.

### Statistical analysis

All data presented in this study were expressed as means ± standard deviation (SD). The statistical analysis was conducted by one-way analysis of variance (ANOVA) to authenticate the validity of results. Duncan’s Multiple Range Test (DMRT, *α* *=* *0.01*) was performed using SPSS program (SPSS, Chicago, IL, USA), to test the statistical significance. Letters on the graph show the result of DMRT (α = 0.01); different letter refer to significant differences between mean values. Student’s t-test (two tailed) was performed to identify statistical significance at two levels, *P < 0.05; **P < 0.01 for suitable data sets.

## Additional Information

**How to cite this article**: Salvi, P. *et al*. Differentially expressed galactinol synthase(s) in chickpea are implicated in seed vigor and longevity by limiting the age induced ROS accumulation. *Sci. Rep*. **6**, 35088; doi: 10.1038/srep35088 (2016).

## Supplementary Material

Supplementary Information

## Figures and Tables

**Table 1:** Biochemical characteristics of CaGolS1 and CaGolS2.

Enzyme	*K*_m_		*V*_max_		pH optimum	Temp optimum
	UDP-Galactose (mM)	*Myo*-inositol (mM)	UDP-Galactose (nmoles mg^−1^ min^−1^)	*Myo*-inositol (nmoles mg^−1^ min^−1^)		
CaGolS1	2.85	23.64	1655.55	1283.33	7.5	40 °C
CaGolS2	3.13	45.5	1703.33	1894.44	7.5	30 °C
