# Supplementary material for: Differentially expressed galactinol synthase(s) in chickpea are implicated in seed vigor and longevity by limiting the age induced ROS accumulation
Source: Sci Rep. 2016 Oct 11;6:35088. doi: 10.1038/srep35088 (PMC5057127; doi:10.1038/srep35088)
Supplement: Supplementary Information [file srep35088-s1.pdf]

Supplementary Information

Differentially expressed galactinol synthase(s) in chickpea are implicated in seed vigor and longevity by limiting the age induced ROS accumulation

Authors

Name: Prafull Salvi, Saurabh Chandra Saxena, Bhanu Prakash Petla, Nitin Uttam Kamble, Harmeet Kaur, PoojaVerma, Venkateswara Rao, Shraboni Ghosh and Manoj Majee*

Affiliation: Lab 203, National Institute of Plant Genome Research,

Aruna Asaf Ali Marg, New Delhi 110067, India.

*Corresponding Author

Name and address of the corresponding author

Name: Dr. Manoj Majee

Address: National Institute of Plant Genome Research, Aruna Asaf Ali Marg, New Delhi-110067, India.

Telephone number: 91-11-26735193

Fax: 91-11-26741658

Email: manojmajee@nipgr.ac.in

Table of Contents:

Figure S1: Photograph of various stages of seed development in chickpea.
Figure S2: Chickpea *GolS* gene sequences with transcript variants.
Figure S3: Native molecular weight determination of CaGolS1 and CaGolS2 protein.
Figure S4: qRT-PCR analysis of *CaGolS1* and *CaGolS2* transcript in respective transgenic lines
Figure S5: GolS activity of *CaGolS1* and *CaGolS2* transgenic lines.
Figure S6: Effect of CDT at 45°C and 100% RH on seed germination.
Figure S7: Effect of raffinose on seed germination after CDT.
Table S1: List of primers.

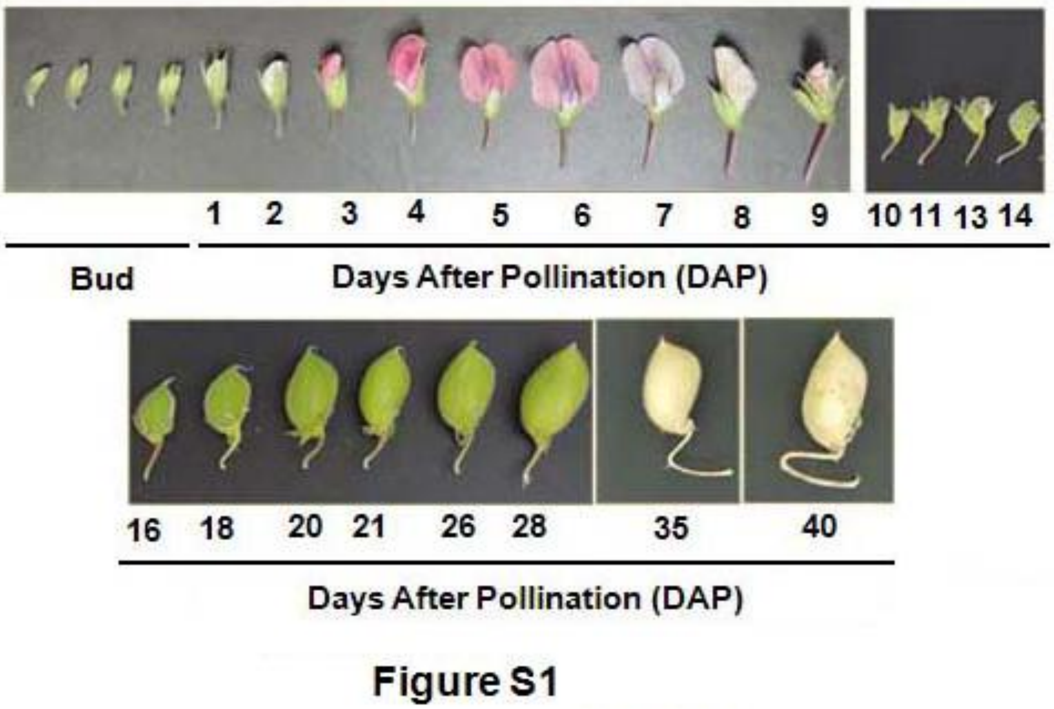

Figure S1: Photograph of various stages (0 to 40 Days after Pollination) of seed development in chickpea.

CaGolS1

Genomic sequence(ATG-TAG)1702 bp

ATGGCTCCAGAACTTGTTCCAACCGCAGCAAAATCAGTTACCGGGTTCACGAAGCCCGCGACTATACCAAAACGTGC
ATTCGTAACATTTCTTGCCGGTAACGGTGATTACGTGAAAGGCGTAATTGGTCTTGCCAAAGGTTTACGGAAGGTGA
AAACGGCGTATCCGCTGGTGGTAGCCGTGCTTCCAGATGTACCGGAGGAACACCGTGAGATGTTGGAGTCTCAGGGA
TGTATTGTCCGTGAGATTGAACCCGTTTACCCACCCGAAAATCAAACCCAGTttGCTATGGCTTATTACGTCATCAA
TTATTCcAAACTCCGTATATGGGAGgtacactaaacactattatactttctacacgtcatcaattttattactatct
ctatgtttacatgtggaatttctgagtcggtttgtttcttacacgtggatccatgcatatgacgtgtcacttactag
acagaaacaaatttaattaatttaactcagtaaatataagttagatggtgaagaactgtgacatggtaccataatc
gaaagaaagtattcattactcattacacaataataaaatcaatttaaaatgatcctagctattaatttgagattgaa
aaattattaaactatcaacaaaattattttattaagaataatgtgcacattcaagaacgcattcagttttgaggctg
catatgcaatcccgtatattgtcttaatgtttaaaaatgaaagttacttttgcgaatagaagaaagtgatttatctt
aaattggaattatgttgcagTTTGTGGAATACAGCAAGATGATATACTTGGACGGAGACATTCAAGTTTATGAAAAC
ATAGATCATCTCTTTGATCTACAAGATGGTTACTTATACGGTGTGATGGATTGTTTCTGCGAGAAAACATGGAGTCA
CACGCCACAATACAAAATTGGTTACTGTCAACAGTGTCCAAATAAGGTACAATGGCCAAAGGAAATGGGTCAACCTC
CTTCACTTTACTTCAATGCTGGCATGTTTGTGTTTGAACCAAGCATTGAAACTTACCATGATCTTTTGAACACACTT
CAAGTCACTCCTCCTACTCCATTTGCAGAACAAGATTTCTTGAACATGTATTTCAAGGATATTTATAAACCAATTCC
TTTGGATTATAACCTAGTTCTTGCTATGTTGTGGCGTCACCCTCAAAATGTTGAGCTTCGGAAAGTCAAGGTTGTTC
ACTATTGTGCAGCGgtatgtatattttaaaattttcccaaatttttacaaattacaatatattaattgtcatgtgt
gacgtgtgacaataactttaattataattaaaatcatttttttaaatattttgacacaatataaattatgaattatt
ttgctaattaataatttatttattgattgtgaattaattataggacatgaattgatttttatttatttattgtttt
tggttaagGGATCAAAGCCTTGGAGATATACAGGGAAGGAAGAGAATATGCAGAGGGAGGACATAAAAATGTTGGTG
CAGAAATGGTGGGATGTTTACAATGACTCTTCACTTGACTATAGCAAGAGCTTGAATGGAAGTAGTGAAACACAAAG
GAATGATGTTGAAAATGAGCCATTCGTACATGCGTTGTCGGAGGTTGGTCATGTTCAATACGTCACTGCCCCTTCAG
CAGCTTAG

CaGolS1 (1020bp) (Accession no KU189226)

ATGGCTCCAGAACTTGTTCCAACCGCAGCAAAATCAGTTACCGGGTTCACGAAGCCCGCGACTATACCAAAACGTGC
ATTCGTAACATTTCTTGCCGGTAACGGTGATTACGTGAAAGGCGTAATTGGTCTTGCCAAAGGTTTACGGAAGGTGA
AAACGGCGTATCCGCTGGTGGTAGCCGTGCTTCCAGATGTACCGGAGGAACACCGTGAGATGTTGGAGTCTCAGGGA
TGTATTGTCCGTGAGATTGAACCCGTTTACCCACCCGAAAATCAAACCCAGTttGCTATGGCTTATTACGTCATCAA
TTATTCcAAACTCCGTATATGGGAGTTTGTGGAATACAGCAAGATGATATACTTGGACGGAGACATTCAAGTTTATG
AAAACATAGATCATCTCTTTGATCTACAAGATGGTTACTTATACGGTGTGATGGATTGTTTCTGCGAGAAAACATGG
AGTCACACGCCACAATACAAAATTGGTTACTGTCAACAGTGTCCAAATAAGGTACAATGGCCAAAGGAAATGGGTCA
ACCTCCTTCACTTTACTTCAATGCTGGCATGTTTGTGTTTGAACCAAGCATTGAAACTTACCATGATCTTTTGAACA
CACTTCAAGTCACTCCTCCTACTCCATTTGCAGAACAAGATTTCTTGAACATGTATTTCAAGGATATTTATAAACCA
ATTCCTTTGGATTATAACCTAGTTCTTGCTATGTTGTGGCGTCACCCTCAAAATGTTGAGCTTCGGAAAGTCAAGGT
TGTTCACTATTGTGCAGCGGGATCAAAGCCTTGGAGATATACAGGGAAGGAAGAGAATATGCAGAGGGAGGACATAA
AAATGTTGGTGCAGAAATGGTGGGATGTTTACAATGACTCTTCACTTGACTATAGCAAGAGCTTGAATGGAAGTAGT
GAAACACAAAGGAATGATGTTGAAAATGAGCCATTCGTACATGCGTTGTCGGAGGTTGGTCATGTTCAATACGTCAC
TGCCCCTTCAGCAGCTTAG

CaGolS1 (339aa)

MAPELVPTAAKSVTGFTKPATIPKRAFVTFLAGNGDYVKGVIGLAKGLRKVKTAYPLVVAVLPDVPEEHREMLESQG
CIVREIEPVYPPENQTQFAMAYYVINYSKLRIWEFVEYSKMIYLDGDIQVYENIDHLFDLQDGYLYGVMDCFCEKTW
SHTPQYKIGYCQQCPNKVQWPKEMGQPPSLYFNAGMFVFEPSIETYHDLLNTLQVTPPTPFAEQDFLNMYFKDIYKP
IPLDYNLVLAMLWRHPQNVELRKVKVVHYCAAGSKPWRYTGKEENMQREDIKMLVQKWWDVYNDSSLDY
SKSLNGSSETQRNDVENEPFVHALSEVGHVQYVTAPSAA*

> (CaGolS1’) (801bp) (Accession no KU189227)

ATGGCTCCAGAACTTGTTCCAACCGCAGCAAAATCAGTTACCGGGTTCACGAAGCCCGCGACTATACCAAAACGTGC
ATTCGTAACATTTCTTGCCGGTAACGGTGATTACGTGAAAGGCGTAATTGGTCTTGCCAAAGGTTTACGGAAGGTGA
AAACGGCGTATCCGCTGGTGGTAGCCGTGCTTCCAGATGTACCGGAGGAACACCGTGAGATGTTGGAGTCTCAGGGA

TGTATTGTCCGTGAGATTGAACCCGTTTACCCACCCGAAAATCAAACCCAGTTTGCTATGGCTTATTACGTCATCAA
TTATTCCAAACTCCGTATATGGGAGTTTGTGGAATACAGCAAGATGATATACTTGGACGGAGACATTCAAGTTTATG
AAAACATAGATCATCTCTTTGATCTACAAGATGGTTACTTATACGGTGTGATGGATTGTCTCTGCGAGAAAGCATGG
AGTCACACGCCACAATACAAAATTGGTTACTGTCAACAGTGTCCAAATAAGCGTCACCCTCAAAATGTTGAGCTTCG
GAAAGTCAAGGTTGTTCACTATTGTGCAGCGGGATCAAAGCCTTGGAGATATACAGGGAAGGAAGAGAATATGCAGA
GGGAGGACATAAAAATGTTGGTGCAGAAATGGTGGGATGTTTACAATGACTCTTCACTTGACTATAGCAAGAGCTTG
AATGGAAGTAGTGAAACACAAAGGAATGATGTTGAAACTGAGCCATTCGTACATGCGTtGTCGGAGGTTGGTCATGT
TCAATACGTCACTGCCCCTTCAGCAGCTTAG

Protein seq (266 aa)

MAPELVPTAAKSVTGFTKPATIPKRAFVTFLAGNGDYVKGVIGLAKGLRKVKTAYPLVVAVLPDVPEEHREMLESQG
CIVREIEPVYPPENQTQFAMAYYVINYSKLRIWEFVEYSKMIYLDGDIQVYENIDHLFDLQDGYLYGVMDCLCEKAW
SHTPQYKIGYCQQCPNKRHPQNVELRKVKVVHYCAAGSKPWRYTGKEENMQREDIKMLVQKWWDVYNDSSLDYSKSL
NGSSETQRNDVETEPFVHALSEVGHVQYVTAPSAA*

CaGolS2

Genomic DNA sequences

ATGGCTCCTGATATAGCAACTGCTGCAGCCAACATCAATGACGCTCAAGCTAAGGCTTCAAAACGCGCCTTTGTTAC
CTTCCTTGCCGGAAACGGAGACTACGTCAAAGGTGTTGTTGGTTTAGTCAAAGGTCTTCGTAAAGTCAAAACCATTT
ACCCTCTAGTGGTTGCAGTGTTACCTGATGTTCCTCAGGAGCACCGGAATATTTTAACTTCTCAAGGTTGTATTGTT
AGAGAGATTCAACCTGTTTACCCTCCTGAGAATCAGACTCAGTTTGCTATGGCTTATTATGTCATCAACTACTCCAA
GCTTCGTATTTGGGCTgtaagaaatctttctattatcaacttttattttaatgttaattttgcattgcatgaatgca
gaggttgcatactctaaagtagttatttgcatggtgtaacatctccaagatacttgtatctagagagatacaaattt
tatctttttttgaactaagagtttgtctctctaaatctaactgttcacacaactgctgcatttttttaaactgttcat
ttagtcataatgtaaaatgttttacaatctaggaaaattagttggtgtaagtttgatttgtgattatgtgttgatcg
taaaatgttgcagTTTGAGGAATATGACAAGATGATTTACCTTGACGGTGACATACAAGTATTTGAAAACATTGACC
ATTTATTTGACCTCCCTAATGACTATTTCTACGCGGTGATGGATTGTTTTTGTGAGGCTACTTGGGGACACACCAAG
CAGTATGAAATCGGTTACTGTCAGCAGTGTCCTGATAAGGTTCAATGGCCCACTAATTTTGGTGCCAAGCCTCCACT
CTACTTCAACGCTGGtTTTTTTGtTTATGAACCTAATATGGCTACTTACCATGATCTTCTTCAAAAACTTAAACTCA
CAAAGCCAACTTCCTTTGCTGAACAGgtacttaattaatcctctaatctttaaatataagaaaaaactattgttgat
ctaacttctcgtatattaagattgacaagaataatcaacttgtttttgtcacttaatatgtatatgtgttttgtag
GATTATTTGAACATGTACTTCAAGGACAAGTATAAGCCTATACCAAATATTTACAATCTTGTGCTGGCTATGCTCTG
GCGTCACCCTGAGAATGTTGAACTTGAGAAAGTCAAAGTGGTTCATTACTGTGCCGCTgtaagttttatttatttga
aagagacaatatagaagttataataaaattggaattttctttaatttggttaacggttatgtgatgatgcagGGGTC
TAAGCCATGGAGGTACACAGGGGTGGAGGAGAATATGGAAAGAGAAGATATAAAGATGTTGGTGAAGAAATGGTGGG
ATATATATGAAGACGAGAGTTTGGATTACAAGGAACCCGTGAATGCGAATCGCTTAACATCAGCACTTTTGGAAGCT
CCTGGTATCAACTTTGTTCCAGCTCCATCAGCCGCTTGA

CaGolS2 (978bp) (Accession no KU214571)

ATGGCTCCTGATATAGCAACTGCTGCAGCCAACATCAATGACGCTCAAGCTAAGGCTTCAAAACGCGCCTTTGTTAC
CTTCCTTGCCGGAAACGGAGACTACGTCAAAGGTGTTGTTGGTTTAGTCAAAGGTCTTCGTAAAGTCAAAACCATTT
ACCCTCTAGTGGTTGCAGTGTTACCTGATGTTCCTCAGGAGCACCGGAATATTTTAACTTCTCAAGGTTGTATTGTT
AGAGAGATTCAACCTGTTTACCCTCCTGAGAATCAGACTCAGTTTGCTATGGCTTATTATGTCATCAACTACTCCAA
GCTTCGTATTTGGGCTTTTGAGGAATATGACAAGATGATTTACCTTGACGGTGACATACAAGTATTTGAAAACATTG
ACCATTTATTTGACCTCCCTAATGACTATTTCTACGCGGTGATGGATTGTTTTTGTGAGGCTACTTGGGGACACACC
AAGCAGTATGAAATCGGTTACTGTCAGCAGTGTCCTGATAAGGTTCAATGGCCCACTAATTTTGGTGCCAAGCCTCC
ACTCTACTTCAACGCTGGTTTTTTTGTTTATGAACCTAATATGGCTACTTACCATGATCTTCTTCAAAAACTTAAAC
TCACAAAGCCAACTTCCTTTGCTGAACAGGATTATTTGAACATGTACTTCAAGGACAAGTATAAGCCTATACCAAAT
ATTTACAATCTTGTGCTGGCTATGCTCTGGCGTCACCCTGAGAATGTTGAACTTGAGAAAGTCAAAGTGGTTCATTA
CTGTGCCGCTGGGTCTAAGCCATGGAGGTACACAGGGGTGGAGGAGAATATGGAAAGAGAAGATATAAAGATGTTGG
TGAAGAAATGGTGGGATATATATGAAGACGAGAGTTTGGATTACAAGGAACCCGTGAATGCGAATCGCTTAACATCA
GCACTTTTGGAAGCTCCTGGTATCAACTTTGTTCCAGCTCCATCAGCCGCTTGA

Protein sequence (325aa)

MAPDIATAAANINDAQAKASKRAFVTFLAGNGDYVKGVVGLVKGLRKVKTIYPLVVAVLPDVPQEHRNILTSQGCIV
REIQPVYPPENQTQFAMAYYVINYSKLRIWAFEEYDKMIYLDGDIQVFENIDHLFDLPNDYFYAVMDCFCEATWGHT
KQYEIGYCQQCPDKVQWPTNFGAKPPLYFNAGFFVYEPNMATYHDLLQKLKLTKPTSFAEQDYLNMYFKDKYKPIPN
IYNLVLAMLWRHPENVELEKVKVVHYCAAGSKPWRYTGVEENMEREDIKMLVKKWWDIYEDESLDYKEPVNANRLTS
ALLEAPGINFVPAPSAA*****

***CaGolS2'* (678bp) (Accession no KU214572)**

ATGGCTCCTGATATAGCAACTGCTGCAGCCAACATCAATGACGCTCAAGCTAAGGCTTCAAAACGCGCCTTTGTTAC
CTTCCTTGCCGGAAACGGAGACTACGTCAAAGGTGTTGTTGGTTTAGTCAAAGGTCTTCGTAAAGTCAAAACCATTT
ACCCTCTAGTGGTTGCAGTGTTACCTGATGTTCCTCAGGAGCACCGGAATATTTTAACTTCTCAAGGTTGTATTGTT
AGAGAGATTCAACCTGTTTACCCTCCTGAGAATCAGACTCAGTTTGCTATGGCTTATTATGTCATCAACTACTCCAA
GCTTCGTATTTGGGCTCCAACTTCCTTTGCTGAACAGGATTATTTGAACATGTACTTCAAGGACAAGTATAAGCCTA
TACCAAATATTTACAATCTTGTGCTGGCTATGCTCTGGCGTCACCCTGAGAATGTTGAACTTGAGAAAGTCAAAGTG
GTTCATTACTGTGCCGCTGGGTCTAAGCCATGGAGGTACACAGGGGTGGAGGAGAATATGGAAAGAGAAGATATAAA
GATGTTGGTGAAGAAATGGTGGGATATATATGAAGACGAGAGTTTGGATTACAAGGAACCCGTGAATGCGAATCGCT
TAACATCAGCACTTTTGGAAGCTCCTGGTATCAACTTTGTTCCAGCTCCATCAGCCGCTTGA

Protein sequence (225aa)

MAPDIATAAANINDAQAKASKRAFVTFLAGNGDYVKGVVGLVKGLRKVKTIYPLVVAVLPDVPQEHRNILTSQGCIV
REIQPVYPPENQTQFAMAYYVINYSKLRIWAPTSFAEQDYLNMYFKDKYKPIPNIYNLVLAMLWRHPENVELEKVKV
VHYCAAGSKPWRYTGVEENMEREDIKMLVKKWWDIYEDESLDYKEPVNANRLTSALLEAPGINFVPAPSAA

***CaGolS2''*(657bp) (Accession no KU214573)**

ATGGCTCCTGATATAGCAACTGCTGCAGCCAACATCAATGACGCTCAAGCTAAGGCTTCAAAACGCGCCTTTGTTAC
CTTCCTTGCCGGAAACGGAGACTACGTCAAAGGTGTTGTTGGTTTAGTCAAAGGTCTTCGTAAAGTCAAAACCATTT
ACCCTCTAGTGGTTGCAGTGTTACCTGATGTTCCTCAGGAGCACCGGAATATTTTAACTTCTCAAGGTTGTATTGTT
AGAGAGATTCAACCTGTTTACCCTCCTGAGAATCAGACTCAGTTTGCTATGGCTTATTATGTCATCAACTACTCCAA
GCTTCGTATTTGGGCTGATTATTTGAACATGTACTTCAAGGACAAGTATAAGCCTATACCAAATATTTACAATCTTG
TGCTGGCTATGCTCTGGCGTCACCCTGAGAATGTTGAACTTGAGAAAGTCAAAGTGGTTCATTACTGTGCCGCTGGG
TCTAAGCCATGGAGGTACACAGGGGTGGAGGAGAATATGGAAAGAGAAGATATAAAGATGTTGGTGAAGAAATGGTG
GGATATATATGAAGACGAGAGTTTGGATTACAAGGAACCCGTGAATGCGAATCGCTTAACATCAGCACTTTTGGAAG
CTCCTGGTATCAACTTTGTTCCAGCTCCATCAGCCGCTTGA

Protein Sequence (218aa)

MAPDIATAAANINDAQAKASKRAFVTFLAGNGDYVKGVVGLVKGLRKVKTIYPLVVAVLPDVPQEHRNILTSQGCIV
REIQPVYPPENQTQFAMAYYVINYSKLRIWADYLNMYFKDKYKPIPNIYNLVLAMLWRHPENVELEKVKVVHYCAAG
SKPWRYTGVEENMEREDIKMLVKKWWDIYEDESLDYKEPVNANRLTSALLEAPGINFVPAPSAA*****

Gene	Transcript variants (bp)	Exon -Intron analysis (bp)	Protein (aa)
CaGolS1 1702bp	*CaGolS1*<br>1020	Exon 1: 333(1-333)<br>Intron 1: 457(334-790)<br>Exon 2: 456 (791 – 1246)<br>Intron 2:225(1247-1471)<br>Exon 3: 231(1472-1702)	339
	CaGolS1'<br>801	Exon 1: 333(1-333)<br>Intron 1: 457(334-790)<br>Exon 2: 180 (791 – 970)<br>Intron 2: 219(971-1189)<br>Exon 3 :57(1190-1246)<br>Intron 3:225(1247-1471)<br>Exon 4: 231(1472-1702)	266
CaGolS2 1502bp	*CaGolS2*<br>978	**Exon1:** 324(1-324)<br>**Intron1:** 305(325-629)<br>**Exon 2:** 321(630-950)<br>**Intron 2:**128 (951-1078)<br>**Exon 3:**135(1079-1213)<br>**Intron 3:** 91 (1214-1304)<br>**Exon 4:** 198 (1305-1502)	325
	CaGolS2'<br>678	**Exon 1:** 324(1-324)<br>**Intron 1:** 605(325-929)<br>**Exon 2:** 21(930-950)<br>**Intron 2:**128 (951-1078)<br>**Exon3:**135(1079-1213)<br>**Intron 3:** 91 (1214-1304)<br>**Exon 4:** 198 (1305-1502)	225
	CaGolS2''<br>657	**Exon 1:** 324(1-324)<br>**Intron 1:** 754(325-1078)<br>**Exon 2** :135(1079-1213)<br>**Intron 2:** 91 (1214-1304)<br>**Exon 3:** 198 (1305-1502)	218

Figure S2

Figure S2: Chickpea GolS gene sequences with various transcripts. Nucleotide sequences of *CaGolS1* and*CaGolS2* genes, transcripts and deduced amino acid sequences. The exon and intron/UTR sequences are written in upper and lower case, respectively.gt- ag splice sites, initiation and termination codons are highlighted. Sequence lengths are summarized in the table.

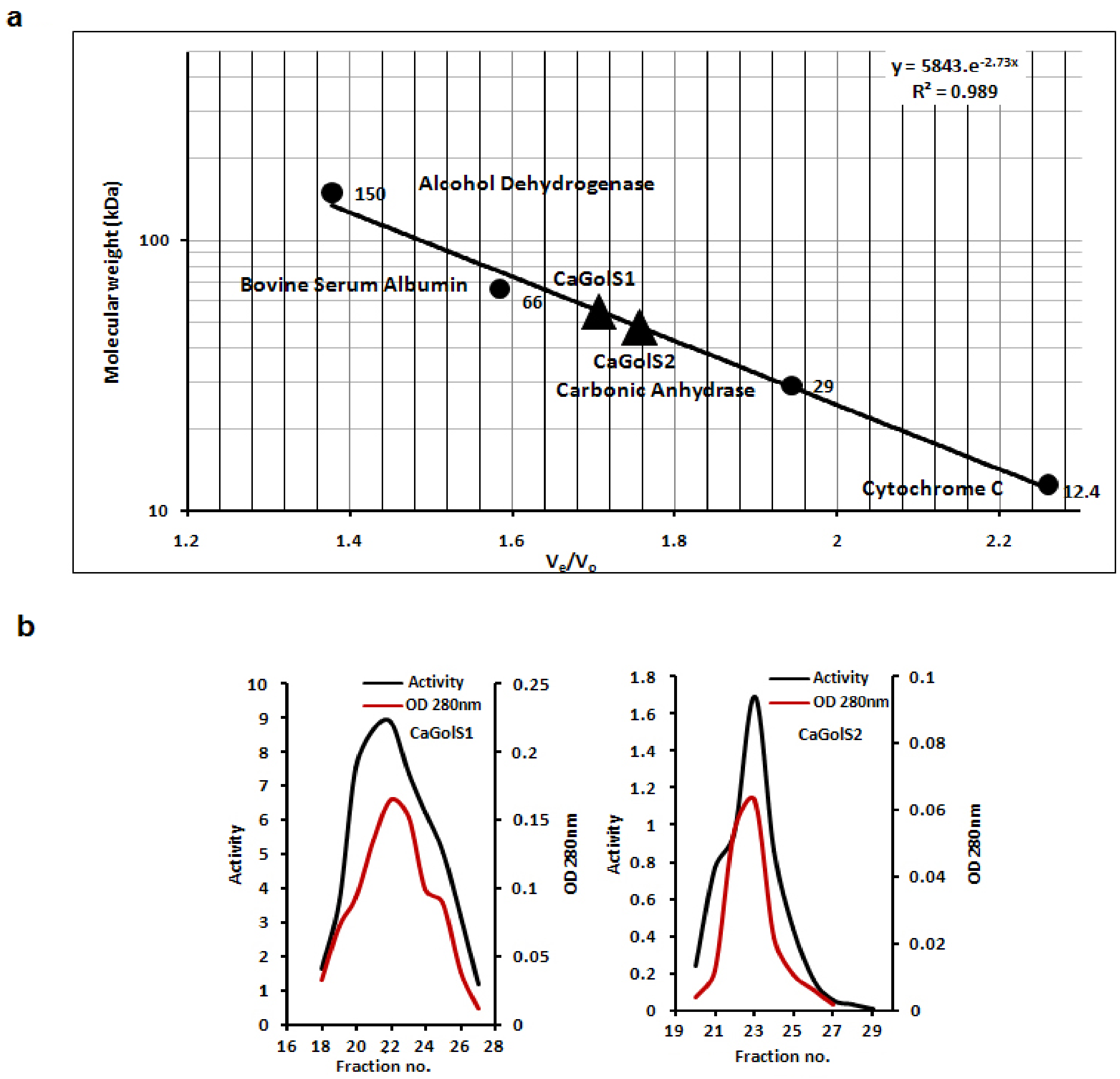

Figure S3

Figure S3. A) Molecular weight determination of CaGolS1 and CaGolS2 through size exclusion chromatography. Purified recombinant proteins were run onto calibrated sephacryl S HR200 gel filtration column.

B) Graphical representation of the GolS activity and A_{280} of collected fractions. Twenty five µl fractions were used for CaGolS assay.

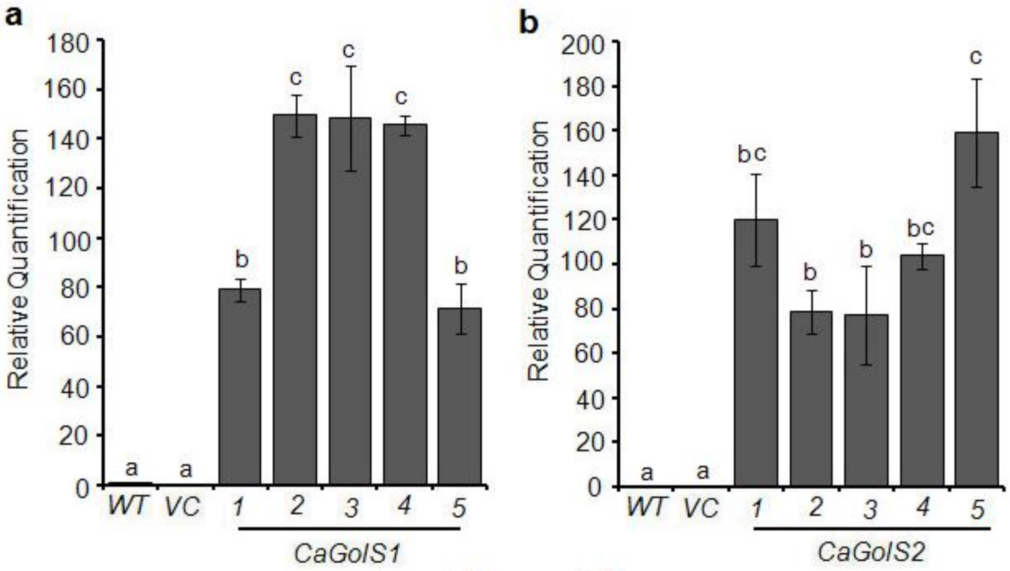

Figure S4

Figure S4. Quantitative RT PCR analysis of *CaGolS*(s) in respective transgenic lines. Total RNA from each sample was reverse transcribed and subjected to real time PCR analysis. The relative expression value of each gene was normalized to an endogenous control 18S and calculated using the$\Delta\Delta$CT method (Applied Biosystems). Values are the result of triplicate analysis of thee biological replicates. Error bars indicate the standard deviation. Significant differences among means (α =0.01) are denoted by the different letters.

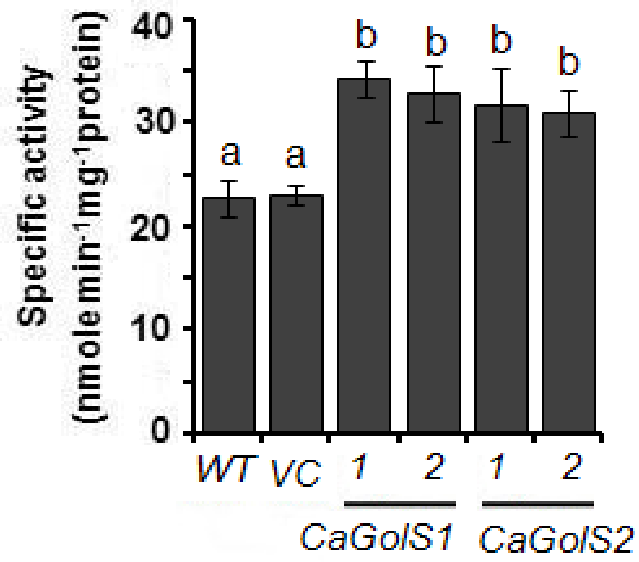

Figure S5

Figure S5. GolS activity was determined in seeds of wild type (*WT*), vector control (*VC*), *CaGolS1*, and *CaGolS2* transformed lines. Fifty µg of crude protein was used for the assay. Specific activity was calculated nanomole Pi (inorganic phosphate) released per mg of protein per min. Data are means ± SD of three biological repeats. Significant differences among means (α =0.01) are denoted by the different letters.

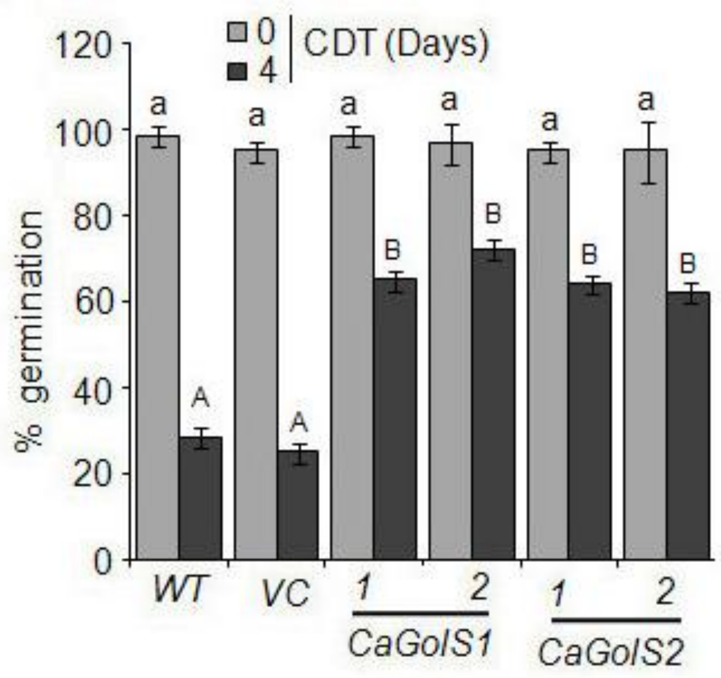

Figure S6

Figure S6. Comparison of germination percentage among seeds of wild type (*WT*), empty vector (*VC*), *CaGolS1,* and *CaGolS2* transformed lines subjected to CDT at 45°C and 100% RH. Experiments on two representative independent transformed lines (For *CaGolS1* 1: L2, 2: L4; For *CaGolS2*1:L1, 2: L5) of each gene are shown here. Eight week old seeds were imbibed to increase moisture content (24% ± 2) and then subjected to CDT for 0 to 4 days (45°C and 100% RH). Germination percentage of wild type (*WT*), empty vector (*VC*), *CaGolS1*, and *CaGolS2* transformed Arabidopsis seeds before and after CDT. Germination was scored after 7 days of imbibition. Data are means ±SD of three biological repetitions with 50 seeds each. Significant differences among means (α =0.01) are denoted by the different letters.

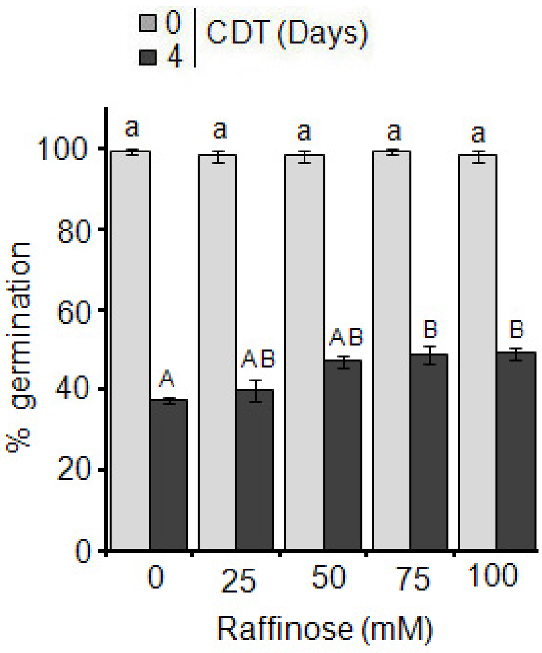

Figure S7

Figure S7. Effect of raffinose on seed germination after CDT. Wild type seeds were subjected to CDT and then were allowed to germinate in presence of increasing concentration of raffinose (0- 100mM). Values are the result of triplicate analysis of thee biological replicates. Error bars indicate the standard deviation. Significant differences among means (α =0.01) are denoted by the different letters.

Table S1 : List of Primer sequences

Name	Sequence 5'→ 3'	Description
MM1 F	AAGCTTCATATGGCTCCAGAACTTGTTCCAACC	Full length/ Bacterial Expression *CaGolS1*
MM2 R	GCTCGAGAGCTGCTGAAGGGGCAGTGA	Full length/ Bacterial Expression *CaGolS1*
MM3 F	AAGCTTCATATGGCTCCTGATATAGCAACTGC	Full length/ Bacterial Expression *CaGolS2*
MM4 R	CTCGAGAGCGGCTGATGGAGCTGGAA	Full length/ Bacterial Expression *CaGolS2*
MM5 F	GCCCGCGACGTTGTGA	qRT- PCR for *Ca18S*
MM6 R	CCTTGTTACGACTTCTCCTTCCTCTA	qRT- PCR for *Ca18S*
MM7 F	TCCACCACTTGGTCGTTTTG	qRT- PCR for *CaEF1α*
MM8 R	CTTAATGACACCGACAGCAACAG	qRT- PCR for *CaEF1α*
MM9 F	CCTCCTTCACTTTACTTCAATGCTG	qRT- PCR for *CaGolS1*
MM10 R	GTTCTGCAAATGGAGTAGGAGGAGT	qRT- PCR for *CaGolS1*
MM11 F	CAACAGTGTCCAAATAAGCGTCACC	qRT- PCR for *CaGolS′*
MM12 R	TCTGCATATTCTCTTCCTTCCCTG	qRT- PCR for *CaGolS1′*
MM13 F	GGGCTTTTGAGGAATATGACAAG	qRT- PCR for *CaGolS2*
MM14 R	GAAATAGTCATTAGGGAGGTC	qRT- PCR for *CaGolS2*
MM15 F	CGTATTTGGGCTCCAACTTCC	qRT- PCR for *CaGolS2′*
MM16 R	TGTAAATATTTGGTATAGGC	qRT- PCR for *CaGolS2′*
MM17 F	CGTATTTGGGCTGGATTATTTG	qRT- PCR for *CaGolS2″*
MM18 R	TGTAAATATTTGGTATAGGC	qRT- PCR for *CaGolS2″*
MM19 F	GAGCTCATGGCTCCAGAACTTGTTCC	To clone *CaGolS1* in plant expression vector (Seed specific)
MM20 R	GTCTAGACTAAGCTGCTGAAGGGGCAGTG	To clone *CaGolS1* in plant expression vector (Seed specific)
MM21 F	GAGCTCATGGCTCCTGATATAGCAAC	To clone CaGolS2 in plant expression vector (Seed specific)
MM22 R	TCTAGATCAAGCGGCTGATGGAGCTG	To clone CaGolS2 in plant expression vector (Seed specific)
MM23 F	CACCATGGCTCCAGAACTTGTTCCAACC	For entry clone of *CaGolS1*
MM24 R	AGCTGCTGAAGGGGCAGTGA	For entry clone of *CaGolS1*
MM25 F	CACCATGGCTCCTGATATAGCAACTGC	For entry clone of *CaGolS2*
MM26 R	AGCGGCTGATGGAGCTGGAA	For entry clone of *CaGolS2*
